# Temporal Dynamics of VEGFA-Induced VEGFR2/FAK Co-Localization Depend on SHB

**DOI:** 10.3390/cells8121645

**Published:** 2019-12-15

**Authors:** Ilkka Pietilä, Djenolan Van Mourik, Andreas Tamelander, Vitezslav Kriz, Lena Claesson-Welsh, Anders Tengholm, Michael Welsh

**Affiliations:** 1 Department of Medical Cell Biology, Uppsala University, Box 571, 75123 Uppsala, Sweden; Ilkka.pietila@igp.uu.se (I.P.); djenolan@live.nl (D.V.M.); andreas-t-97@hotmail.com (A.T.); anders.tengholm@mcb.uu.se (A.T.); 2 Present address: Department of Immunology, Genetics and Pathology, Uppsala University, 75108 Uppsala, Sweden; 3 Institute of Molecular Genetics of the CAS, 14220 Prague, Czech Republic; vkriz.cz@gmail.com; 4 Department of Immunology, Genetics and Pathology, Uppsala University, 75108 Uppsala, Sweden; lena.welsh@igp.uu.se

**Keywords:** VEGFR2, FAK, SHB, TIRF, focal adhesions, angiogenesis

## Abstract

Focal adhesion kinase (FAK) is essential for vascular endothelial growth factor-A (VEGFA)/VEGF receptor-2 (VEGFR2)-stimulated angiogenesis and vascular permeability. We have previously noted that presence of the Src homology-2 domain adapter protein B (SHB) is of relevance for VEGFA-stimulated angiogenesis in a FAK-dependent manner. The current study was conducted in order address the temporal dynamics of co-localization between these components in HEK293 and primary lung endothelial cells (EC) by total internal reflection fluorescence microscopy (TIRF). An early (<2.5 min) VEGFA-induced increase in VEGFR2 co-localization with SHB was dependent on tyrosine 1175 in VEGFR2. VEGFA also enhanced SHB co-localization with FAK. FAK co-localization with VEGFR2 was dependent on SHB since it was significantly lower in SHB deficient EC after VEGFA addition. Absence of SHB also resulted in a gradual decline of VEGFR2 co-localization with FAK under basal (prior to VEGFA addition) conditions. A similar basal response was observed with expression of the Y1175F-VEGFR2 mutant in wild type EC. The distribution of focal adhesions in SHB-deficient EC was altered with a primarily perinuclear location. These live cell data implicate SHB as a key component regulating FAK activity in response to VEGFA/VEGFR2.

## 1. Introduction

Angiogenesis is the process by which new vessels are formed on demand and this response is of importance for numerous (patho)physiological conditions, such as wound healing, the menstrual cycle and inflammation. Vascular endothelial growth factor-A (VEGFA) is the main angiogenic factor and influences numerous endothelial cell (EC) mechanisms, such as proliferation, migration, vascular leakage and in vivo angiogenesis [1,2,3]. The VEGF-receptor2 (VEGFR2) signals mainly via three main phosphorylation sites: Y951, Y1175 and Y1214, and their phosphorylation generates binding sites for SH2D2A (Y951) and phospholipase C-gamma (1175) [4]. Y1214 is thought to mediate VEGFA-dependent c-Myc activation [5]. Consequently, binding of these components to their respective binding sites will initiate downstream signaling, resulting in extracellular-signal regulated kinase (ERK), phosphatidylinositol 3′-kinase (PI3K) and Rho-family GTPase activation. One essential signaling intermediate is focal adhesion kinase (FAK) that is required for VEGFA-induced angiogenesis and vascular permeability [6,7]. The details on how VEGFA regulates FAK activity remain, however, elusive.

SHB is an adapter protein [8] of relevance for VEGFA-induced angiogenesis and vascular permeability [9,10,11,12,13,14]. SHB interacts with FAK [15], FAK phosphorylates SHB [16] and in vitro protein interaction studies demonstrate that tyrosine 1175 in VEGFR2 is a SHB SH2-domain binding site that conveys, at least partly, VEGFA-dependent FAK activation [17]. The in vivo relevance of this response, however, remains unresolved. The *Shb* knockout (KO) mouse shows in numerous cell types an increase in basal FAK activity [11,14,18,19,20] and loss of VEGFA-stimulated FAK activity [11,14]. Consequently, EC spreading is increased under basal conditions, without VEGFA addition causing EC to spread further [11], whereas EC migration is diminished in response to VEGFA [14]. Although the loss of ligand-induced FAK activity is in line with a role of SHB in conveying receptor-dependent FAK signaling, the elevated basal FAK activity observed in the absence of SHB has hitherto remained unexplained. Regardless, the data suggest that SHB is an intermediate in VEGFA-dependent FAK activation that ultimately plays a key role in angiogenesis and vascular leakage.

The study was performed to gain a deeper understanding of the molecular mechanisms behind the role of SHB in VEGFA-dependent FAK activation. Total internal reflection fluorescence microscopy (TIRF) was employed to visualize sub-membranous co-localization of fluorescently tagged VEGFR2, SHB and FAK in response to VEGFA. These novel data based on live-cell recordings demonstrate a role of SHB in the normal temporal dynamics of VEGFR2 and FAK co-localization in EC.

## 2. Materials and Methods

### 2.1. Mice

Wild type (+/+) and *Shb* knockout (KO; −/−) [21]] Balb/c mice housed at the animal department of the Biomedical Centre at Uppsala University were used for preparations of primary lung endothelial cells. All animal experiments were approved by the county animal ethics committee regulating animal housing at Uppsala University (approval number C22/14).

### 2.2. DNA Constructs

The mCherry (when co-transfected with SHB) and mEmerald (when co-transfected with VEGFR2) FAK (*PTK2*) constructs were purchased from Addgene (Watertown, MA, USA, Addgene.org; #55122 and #54241). The eGFP-SHB construct was generated by inserting the human *SHB* cDNA starting with the initiator ATG in frame downstream of the eGFP sequence with a linker in the pEGFP-N1 vector. The *SHB* cDNA included 137 nucleotides downstream of the stop codon. The linker sequence (underlined) between eGFP and *SHB* was TACAAGTCCATGGCC.

The mCherry-VEGFR2 pcDNA3.1 construct contained VEGFR2 cDNA inserted into the KpnI site followed by a linker, and in frame with the mCherry sequence. The linker sequence was GGTGGGAGTGGAGGTGGGAGTGGA. The wild type receptor construct was used as a template for the mutants. The KpnI-AgeI receptor fragment was replaced with the corresponding mutant fragments that were obtained from previously generated cDNAs [22]. All constructs were sequence verified.

### 2.3. Cell Isolation and Transfection

Mouse lungs were collected from self-bred neonatal wild type and *Shb* KO Balb/c mice at P8–10 or 3–4 weeks. Excised lungs were minced and digested into a single cell suspension in 10 mL Dulbecco’s PBS medium containing 2 mg mL^−1^ collagenase type I (Sigma-Aldrich, St Louis, MO, USA, #C0130), for 1 h at 37 °C with rotation, followed by filtration through a 70 μm cell strainer (BD Falcon, Schaffhausen, Switzerland) in a manner similar to that described in [14]. Cells were centrifuged at 400× *g* for 8 min at 4 °C and suspended in cold PBS/0.1% bovine serum albumin (BSA). The cells were then incubated with biotin-CD31 antibody (Biolegend, San Diego, CA, USA, #102504) for 15 min with gentle agitation on ice and washed with 1 mL PBS/0.1% BSA, followed by a 300× *g* spin for 10 min. The pellet was resuspended in 200 µL beads buffer (PBS/0.1% BSA/2 mM EDTA) and 5 µL Anti-Biotin Microbeads (Miltenyi Biotec, Bergisch Gladbach, Germany #130-090-485) was added. The suspension was incubated for 15 min with gentle agitation on ice and washed with 1 mL beads buffer at 300× *g* for 10 min. During the centrifugation, MACS Cell Separation Columns (Miltenyi Biotec, Bergisch Gladbach, Germany #130-042-201) were prepared in a MiniMACS separator (Miltenyi Biotec, #130-042-303) and rinsed with 500 µL beads buffer. The pellet was suspended in 500 µL beads buffer and loaded onto the column and washed three times with 500 µL beads buffer. Cells were eluted to 1 mL of MV2 growth medium (EC medium) with the following supplements: 5 ng mL^−1^ endothelial growth factor; 0.2 μg mL^−1^ hydrocortisone; 0.5 ng mL^−1^ VEGFA; 10 ng mL^−1^ basic fibroblast growth factor; 20 ng mL^−1^ insulin-like growth factor-1 (Promocell, Heidelberg, Germany, #C-39221); and 5% penicillin/streptomycin (Sigma, #P0781). The cells were cultured in a 24-well plate, coated with fibronectin. After plating, 75% ± 5% (*n* = 4) of the cells were CD31 positive and among the cells that showed a spread, flattened morphology (which was the criterium for further TIRF analysis), virtually all were CD31 positive. The cells were cultured 50–60% confluent and transfected with Lipofectamin 3000 (ThermoFisher, Waltham, MA, USA, #L3000001) according to the manufacturer’s instructions. HEK293 cells were cultured in DMEM with 10% fetal bovine serum (FBS) and antibiotics. Transfection efficiency was low and attempts to generate cell lines stably overexpressing VEGFR2 consistently failed, precluding biochemical analysis of the transfected products. In addition, each viable cell expressing the tagged proteins exhibited a low to moderate degree of overexpression.

### 2.4. TIRF Microscopy

TIRF microscopy was essentially done as described in [23]. Transfected EC or HEK293 cells on fibronectin coated coverslips were perifused in EC medium without growth factor supplements for EC or 7.305 g NaCl, 0.365 g KCl, 0.264 g MgCl_2_, 0.176 g CaCl_2_, 100 mL ddH2O, 11.92 g HEPES (pH = 7.4) per 100 mL for HEK293 cells and subjected to imaging with a Nikon Eclipse Ti microscope, Nikon Corp, Minato, Japan (100× oil objective, NA 1.49) equipped with a TIRF illuminator and lasers exciting at 491 and 561 nm. The illuminator angle was set to promote maximal total internal reflection. Interference filters (Semrock, Rochester, NY, USA) used were 530/50 nm half-bandwidth and 620 longpass filter in a filter wheel (Sutter Instruments, Novato, CA, USA). For detection, a back-illuminated EMCCD camera (DU897, Andor Technology, Belfast, UK) controlled by MetaFluor software (Molecular Devices Corp, Downington, PA, USA) was used. Dual wavelength images were collected every 5 s. The cells were recorded for 5 min under basal conditions followed by a 5-min stimulation period with 100 ng mL^−1^ of VEGFA. For analysis in ImageJ, a raster grid consisting of 144 raster points (12 × 12) was placed over an area of interest outside the nucleus in a cell that had spread out. Due to spreading, these EC were very thin and thus the nucleus could be identified as an area with a lower fluorescence intensity. The raster area chosen for analysis was kept the same throughout the experiment and each raster point corresponded to four pixels (260 nm × 260 nm). For each raster point the signals in the eGFP/mEmerald and mCherry channels were determined synchronously for each time point. When the eGFP/mEmerald channel co-localization with the mCherry channel was assessed, raster points in which the mCherry signal was above the mean mCherry signal at each point of measurement were selected and the corresponding total eGFP/mEmerald channel signal for those raster points was calculated and determined as a fraction of the total eGFP/mEmerald channel signal in all raster points at that time point. The converse was done when mCherry co-localization with eGFP/mEmerald was determined since co-localization of one of the tagged proteins was not always reciprocated when co-localization was determined in the other direction. In the main figures, each recording was used as one observation for determining the trendlines (slopes) for the intervals and comparisons could thus be made between before and after VEGFA addition. In the Supplemental Figures, means of the values for all recordings at each time point was calculated and trendlines (slopes) were determined based of those means, thus explaining the discrepancy between the numerical values in the main and Supplemental Figures. Please note that the changes in trendlines/slopes cannot be detected visually (see Supplemental Movie 1) but only by numerical analysis in Excel of co-localization data. Absolute values of co-localization, unlike the changes in trendlines/slopes before and after VEGFA, showed large experimental variations as apparent from the Supplemental Figures and were thus not analyzed.

### 2.5. Paxillin Staining

Non-transfected EC cells grown on fibronectin coated coverslips were fixed with cold 4% PFA for 15 min at RT and washed 3× 5 min with PBS. Unspecific binding was blocked with 10% FBS/PBS for 1h at RT. Anti-paxillin antibody (BD Biosciences, San Jose, CA, USA, #610619) was diluted in 1% FBS/PBS and incubated overnight at 4 °C. Cells were washed 3× 5 min with PBS and the incubated with donkey anti-mouse AlexaFluor 594 (Invitrogen, Waltham, MA, USA, www.Thermofisher.com) secondary antibody for 1 h at 4 °C, followed by 3× 5 min washes with PBS and PBS with 1 μg mL^−1^ Hoechst 33342 for 15 min, followed by a quick wash and mounting on an objective glass. Confocal images of EC that had spread were taken and analyzed by ImageJ for fluorescence intensity in the peripheral area vs. the perinuclear area. The boundary between the perinuclear and peripheral was set at half the distance from the nucleus to the edge of the cell and the fluorescence intensity in each region determined. Ratios were calculated for each cell (10 wild type and 8 KO).

### 2.6. Statistical Analysis

Trendlines (every third observation point) were determined in Excel and compared against the basal trendline for stimulated values or for the basal trendlines compared against zero change by Student’s t-tests. The basal trendline was normally based on the 2.5 min preceding stimulation unless the signal showed major fluctuations during that period. Under such circumstances, an extended period was chosen for assessing the basal trendline. Means ± SEM for the number of observations are given.

## 3. Results

### 3.1. SHB and VEGFR2 Co-Localization

**Figure 1:**
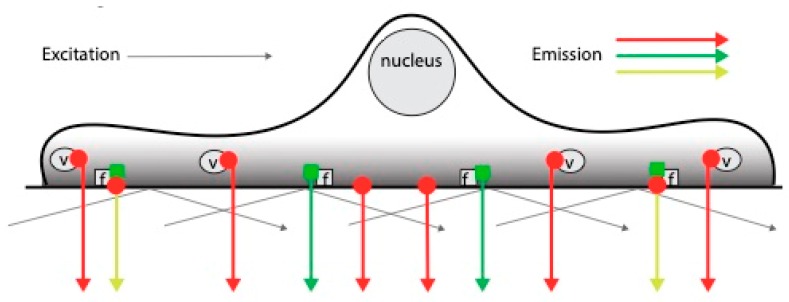
Hypothetical model illustrating patterns of TIRF emission depending on the co-localization between probe A (red) and probe B (green) having different subcellular localizations with limited overlap. The shaded gray area indicates the TIRF zone. v indicates intracellular vesicles and f focal adhesions. Depending on the stimulus/conditions, the yellow signal (co-localization) will vary.

To address the temporal dynamics of SHB/VEGFR2 co-localization, HEK293 cells were transfected with eGFP-SHB and mCherry-VEGFR2 (wild type and 951F, 1175F and 1214F mutants) since endogenous VEGFR2 expression in EC would interfere with the results of such studies. The data are trendlines (= slopes) of the plots based on TIRF recordings (Supplemental Movie 1 gives an example of such a recording) showing changes in rates of co-localization in the sub-membranous space with time (Supplemental Figures S1–3). Figure 1 depicts TIRF in a hypothetical scenario with probe A (red) and probe B (green) mainly located in separate subcellular compartments with limited overlap. The emitting signals will have a red, green or yellow appearance, depending on the degree of co-localization.

**Figure 2:**
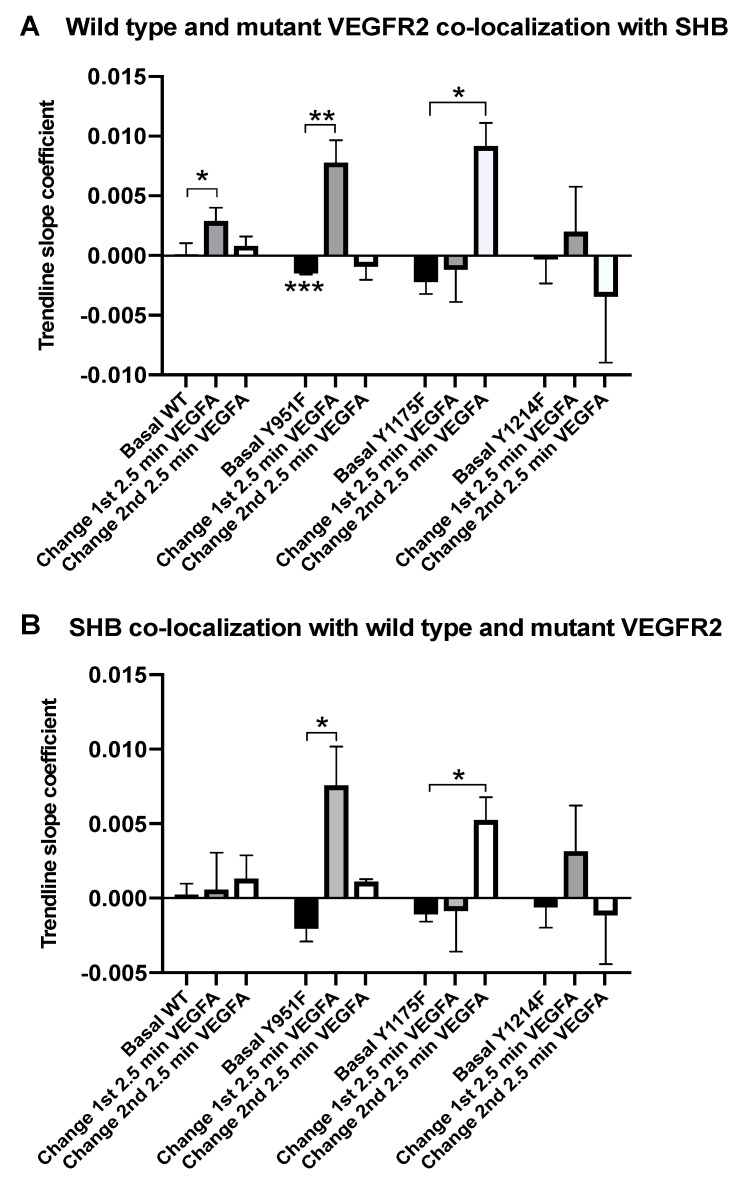
SHB and VEGFR2 co-localization by TIRF microscopy. (**A**) shows VEGFR2 co-localization with SHB and (**B**) SHB co-localization with VEGFR2. HEK293 cells on fibronectin-coated coverslips were transfected with plasmids encoding eGFP-SHB and mCherry-VEGFR2, wild type or Y961F, as well as Y1175F and Y1214F mutants. TIRF signals were recorded for 5 min without addition (basal) prior to 100 ng mL^−1^ VEGFA addition. The stimulated TIRF signal was divided into two 2.5 min periods. TIRF co-localization was determined by calculating the ratio of the co-localized signal over total signal at each time point. Trendlines from such plots were calculated in Excel giving the trendline’s slope coefficient. One TIRF recording yielded one trendline/slope for each co-localization. Values for each wild type or mutant receptor are presented in groups with the basal trendline/slope followed by trendline/slope changes relative to the basal in response to VEGFA for each of the first and second 2.5 min periods. Means ± SEM are given for 4–6 separate observations determined at two transfection occasions. *, ** and *** indicate *p* < 0.05, *p* < 0.01 and *p* < 0.001, respectively, when compared with a Student’s t-test against the zero trendline (basal comparison) or basal trendline (other comparisons).

When analyzing actual TIRF recordings, VEGFR2 exhibited an increased rate of co-localization with SHB during the first 2.5 min of VEGFA addition, an effect that was not detected during the subsequent 2.5 min (Figure 2A). The same pattern after stimulation was also detected in cells expressing the Y951F mutant, although this mutant demonstrated a significant decrease in the rate of VEGFR2 co-localization with SHB during basal conditions, i.e., prior to VEGFA stimulation. The Y1175F mutant, on the other hand, showed a delay in its co-localization with SHB, only becoming apparent subsequent to the initial 2.5 min of stimulation. The Y1214F VEGFR2 mutant displayed no significant differences in association rates although the pattern during the basal and early stimulation periods resembled that of the wild type receptor.

The patterns of SHB co-localization with VEGFR2 resembled that of VEGFR2 co-localization with SHB for the mutant receptors, which is in contrast to the wild type receptor’s response in which no significant differences were observed (Figure 2B). The discrepancy in the reciprocity between wild type VEGFR2/SHB and SHB/wild type VEGFR2 responses may be explained by differences in the subcellular compartmentalization of these components as can be inferred from Figure 1 or, alternatively, different degrees of overexpression after transfection.

The data support the view that the primary binding site for SHB to VEGFR2 is Y1175.

### 3.2. SHB and FAK Co-Localization

**Figure 3:**
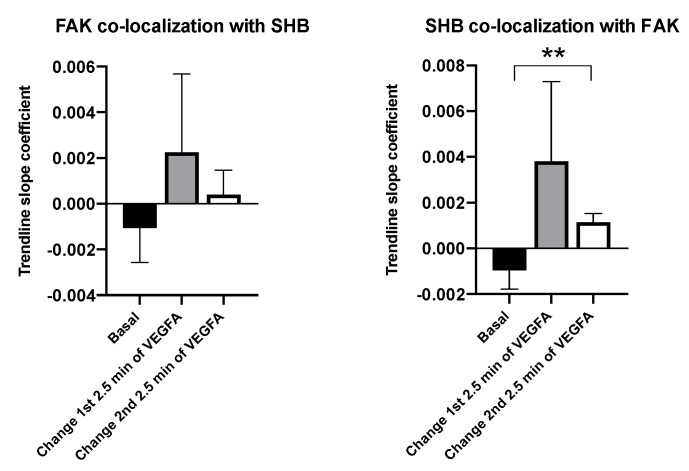
SHB and FAK co-localization by TIRF microscopy in lung endothelial cells (EC). Isolated primary EC growing on fibronectin-coated coverslips were transfected with eGFP-SHB and mCherry-FAK. Trendlines/slopes of co-localization were determined as in Figure 2 with basal (unstimulated) and VEGFA-stimulated values that were separated into the first and second 2.5-min periods after stimulation. Means ± SEM for six observations from four transfection experiments are given. ** indicates s *p* < 0.01 obtained from a Student’s t-test when compared with the basal value.

FAK co-localization with SHB in isolated wild type lung primary lung EC showed no significant changes in response to VEGFA (Figure 3). During the first 2.5 min of VEGFA stimulation a numerical increase in SHB co-localization with FAK was observed that failed to reach statistical significance due large experimental variation. SHB co-localization with FAK was significantly increased during the following 2.5 min of stimulation (Figure 3), indicating that SHB/FAK interplay is of relevance for VEGFA-dependent responses in EC.

### 3.3. VEGFR2 and FAK Co-Localization

**Figure 4:**
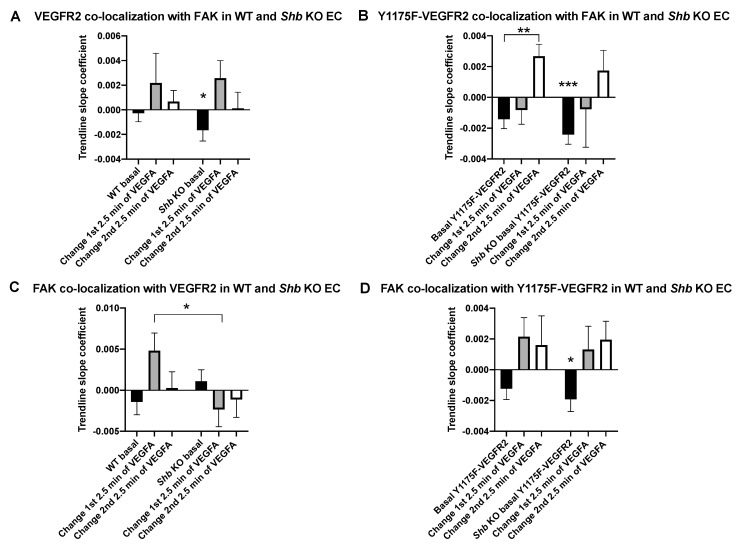
VEGFR2 and FAK co-localization by TIRF microcopy in wild type and *Shb* KO lung EC. Isolated primary EC growing on fibronectin-coated coverslips were transfected with mEmerald-FAK and mCherry-wild type or Y1175F-VEGFR2. (**A**) VEGFR2 co-localization with FAK, (**B**) Y1175F-VEGFR2 co-localization with FAK, (**C**) FAK co-localization with VEGFR2 and (**D**) FAK co-localization with Y1175F-VEGFR2. Values were determined as in Figure 2 and are basal (unstimulated) and VEGFA-stimulated relative basal rates divided into the first and second 2.5 min periods as indicated. Means ± SEM are given for 5–6 observations (wild type receptor) and 10–13 observations (Y1175F-VEGFR2). Two transfection experiments for the wild type receptor and 1–2 transfection experiments for the mutant receptor were done. Student’s t-test were done. * in (**A**) indicates *p* < 0.05 when compared with the zero-change basal. *** in (**B**) indicates *p* < 0.001 when compared with zero change; and ** indicates *p* < 0.01 when compared with basal. In (**C**), * indicates *p* < 0.05 when compared with the corresponding *Shb* KO value. In (**D**), * indicates *p* < 0.05 when compared with the zero basal.

Mutant and wild type VEGFR2 co-localization with FAK was assessed in wild type and *Shb* KO lung EC (Figure 4, Supplemental Figures S4,5). Wild type VEGFR2 showed no significant change in its co-localization with FAK in wild type EC whereas its co-localization decreased under basal conditions prior to VEGFA addition in the *Shb* KO EC (Figure 4A). Y1175F-mutated VEGFR2 exhibited a delayed co-localization with FAK in response to VEGFA that became significant subsequent to the initial 2.5 min stimulation period when tested in wild type EC (Figure 4B). The basal co-localization was decreased in EC deficient in SHB and in these cells VEGFA failed to stimulate co-localization.

When FAK co-localization with VEGFR2 was assessed, the initial increase in co-localization after VEGFA addition observed in wild type EC was completely lost by SHB depletion (Figure 4C). The pattern of FAK co-localization with Y1175F-VEGFR2 showed only a significant decrease in co-localization under basal conditions in *Shb* KO EC (Figure 4D).

The data implicate SHB in FAK/VEGFR2 co-localization and that Y1175 is essential for its early response.

### 3.4. Focal Adhesions in Shb KO EC

**Figure 5:**
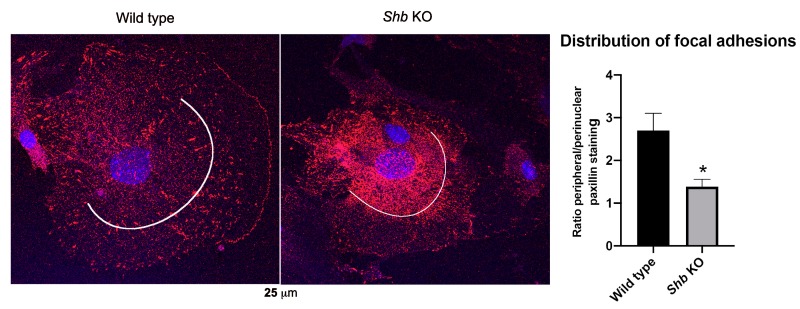
Focal adhesions in wild type and *Shb* KO lung EC. Cells were immunostained for paxillin using a donkey anti-mouse AlexaFluor 594 secondary antibody and the relative intensity of the signal in cells that had spread was separated into the perinuclear and peripheral regions as indicated by the lines and quantified by ImageJ. The column chart indicates the ratios peripheral/perinuclear signal for wild type versus *Shb* KO values for 10 and 8 cells. Means ± SEM are given. * indicates *p* = 0.011 from a Student’s t-test.

To visualize focal adhesions in EC, isolated cells were stained for paxillin (Figure 5). When cells that had spread were examined, focal adhesions showed a primarily peripheral location in wild type cells. Cells deficient in SHB exhibited a more perinuclear location and this difference was statistically significant when fluorescence intensity was analyzed as visualized by the lines in the figures that indicate the separation between peripheral/perinuclear staining. Total fluorescence intensity was not different between wild type and KO EC. Similar patterns were detected when mEmerald-FAK fluorescence was visualized from a TIRF recording in wild type and *Shb* KO EC (Supplemental Figure S6). The pattern of the VEGFR2 signal resembled that previously observed by immunostaining EC for VEGFR2 [14]].

The data indicate changes in focal adhesions occurring as a consequence of SHB deficiency that have an impact on VEGFR2/SHB/FAK signaling.

## 4. Discussion

The study was conducted in order to address the relevance of SHB for VEGFR2/FAK signaling in EC. For this purpose, TIRF microscopy was employed allowing detection of the temporal dynamics of co-localization in the vicinity of the plasma membrane (sub-membranous space). The co-localization data do not necessarily imply direct interactions but could rather reflect their presence in juxtaposed complexes although we have previously reported associations between SHB and VEGFR2 or FAK by fusion protein binding experiments, suggesting indeed that the current results may reflect the formation of complexes containing these components [15,17]. In some experiments the changes in the co-localizations detected were only unidirectional. This could partly reflect statistical aberrations due to large experimental variations in certain settings but could also indicate differences in turnover depending on the subcellular compartment in which the molecule studied is located. VEGFR2 is mainly localized to intracellular vesicles [24] whereas FAK is to a large extent found in focal adhesions [25], and if the interface between these compartments in relative terms is smaller for VEGFR2 than for FAK, a detectable co-localization in the latter case may remain unnoticed for VEGFR2. Figure 1 illustrates this argument. Another possibility is that the relative degree of overexpression might vary between the conditions and that this could influence whether the co-localizations may occur in both directions or not.

The data indicate that SHB is required for the normal dynamics of VEGFR2/FAK co-localization in response to VEGFA. The most apparent finding indicating this was the significant reduction in FAK/VEGFR2 co-localization between wild type and *Shb* KO EC. Another finding relating to the *Shb* KO phenotype was the reduction in basal VEGFR/FAK co-localization. Both these findings are compatible with SHB serving a role as an intermediary between VEGFR2 and FAK.

The dynamics of VEGFR2/SHB and VEGFR2/FAK co-localization suggest Y1175 as a significant binding site for the SHB SH2 domain as previously reported [17]. However, a delayed response was observed, and this allows two possible interpretations: Additional binding sites for SHB exist in VEGFR2 that respond more slowly or that signaling complexes are slowly generated in the absence of tyrosine 1175 that include SHB, directly or indirectly. Additional findings supporting a role of tyrosine 1175 in SHB dependent signaling are the decreased co-localization rates between Y1175F-VEGFR2 and FAK, which mirror the finding with the wild type receptor in *Shb* KO cells, indicating that SHB plays a role in this process by interacting with tyrosine 1175 regardless of whether the receptor has been stimulated or not. Possible explanations for the effect in the absence of VEGFA are residual receptor tyrosine phosphorylation or altered intracellular compartmentalization of these components in the absence of SHB.

Studies demonstrate that receptor activation and tyrosine phosphorylation will cause recruitment and clustering of signaling complexes that depend on their relative affinities to receptor phosphotyrosine sites and expression levels [26,27]. Receptor mutations will rewire these signaling complexes [26,27] and the current data with the Y1175F mutation thus reflects such a phenomenon. However, we argue that even though SHB eventually co-localizes with Y1175F-VEGFR2, this reflects altered temporal dynamics and composition of the assembled signaling complex, which has severe consequences for the cellular response to VEGFA as has been well established [28].

The Y951F-VEGFR2 mutant displays unexpected features. These include reduced basal association rates and accentuated co-localization rates after stimulation. As mentioned above for Y1175F, the most likely explanation lies in the generation of aberrant signaling complexes upon stimulation of this mutant receptor that more rapidly disassemble in the absence of ligand.

Focal adhesions redistributed from the periphery towards a more perinuclear region in the absence of SHB and this is likely to have an influence on the dynamics of VEGFR2/FAK co-localization. Whether the redistribution is a response to the altered VEGFR2 signaling occurring in the absence of SHB or due to other effects of SHB deficiency, the data do not reveal. However, active VEGFR2 promotes FAK localization to adherens junctions and VE-cadherin [6], an effect necessary for VEGFA-induced vascular permeability, and consequently re-localization of focal adhesions to the perinuclear region is likely to impair that process. Furthermore, it has been demonstrated that EC expressing the Y397F-FAK mutant display fewer peripheral focal adhesions [29], suggesting indeed that aberrant FAK signaling underlies our currently demonstrated focal adhesion phenotype in SHB-deficient EC. However, alternative possibilities can be envisaged. SHB has been demonstrated to operate downstream of other tyrosine kinase receptors, such as the fibroblast growth factor receptor-1 (FGFR1) [30], and this receptor also plays a role for EC biology. Thus, the findings on altered focal adhesion morphology could reflect aberrant FGFR1 signaling that disturbs some process related to focal adhesion assembly, with secondary consequences for VEGFR-SHB-FAK signaling as currently demonstrated.

## 5. Conclusions

The live cell data demonstrate a role of SHB in the temporal dynamics of VEGFR2-dependent FAK signaling, which parallels altered distribution of focal adhesions in EC. These alterations are strong candidates for explaining the deficient EC phenotype in Y1175F-VEGFR2 and *Shb* null EC.

## Supplementary Materials

The following are available online at https://www.mdpi.com/2073-4409/8/12/1645/s1, Supplemental Figure S1: TIRF co-localization as indicated. Co-localization at each time point was determined for each recording (individual observation) and means ± SEM for the individual time point values were calculated and plots generated for each experimental condition. These were used to obtain trendlines (trendlines = slopes) that are shown with values indicated in the legends. These will be different from the ones shown in Figure 2, Figure 3 and Figure 4 since the latter are means of individual trendlines/slopes, each obtained from a separate recording, *n* = 6. Supplemental Figure S2: TIRF co-localization as described in Supplemental Figure S1 with trendlines (= slopes) indicated, *n* = 6. Supplemental Figure S3: TIRF co-localization as indicated in Supplemental Figure S1 with trendlines (= slopes) indicated, *n* = 6. Supplemental Figure S4: TIRF co-localization as indicated in Supplemental Figure S1. Trendlines (= slopes) were determined as indicated in the legends and will be different from the ones shown in Figure 4 since the latter are means of individual trendlines for each recording whereas the trendlines of this figure are based on pooled co-localization data for each time point, *n* = 6. Supplemental Figure S5: TIRF co-localization as indicated in Supplemental Figure S1. Trendlines (= slopes) are given, *n* = 5. Supplemental Figure S6: TIRF snapshots of mEmerald-FAK (green) and mChreey-VEGFR2 (red) fluorescence after transfection to wild type and *Shb* KO EC. Note the perinuclear localization of mEmerald-FAK in the KO situation that resembles the corresponding paxillin staining pattern whereas the wild type cells display peripheral localization. Supplemental Movie S1: Example of TIRF recording of EC transfected with mEmerald-FAK (green) and mCherry-VEGFR2 (red) (co-localization yellow) for 2.5 min prior to VEGFA addition followed by 2.5 min with VEGFA. Please note that the changes in trendlines cannot be seen visually and only detected by numerical analysis in Excel.
