# Supplementary material for: Temporal Dynamics of VEGFA-Induced VEGFR2/FAK Co-Localization Depend on SHB
Source: Cells. 2019 Dec 15;8(12):1645. doi: 10.3390/cells8121645 (PMC6953046; doi:10.3390/cells8121645)
Supplement: Supplementary file 1 [file cells-08-01645-s001.zip › cells supplementary-655556 revised.pdf]

Supplemental Figure 1

HEK293 cells and SHB/wild type VEGFR2

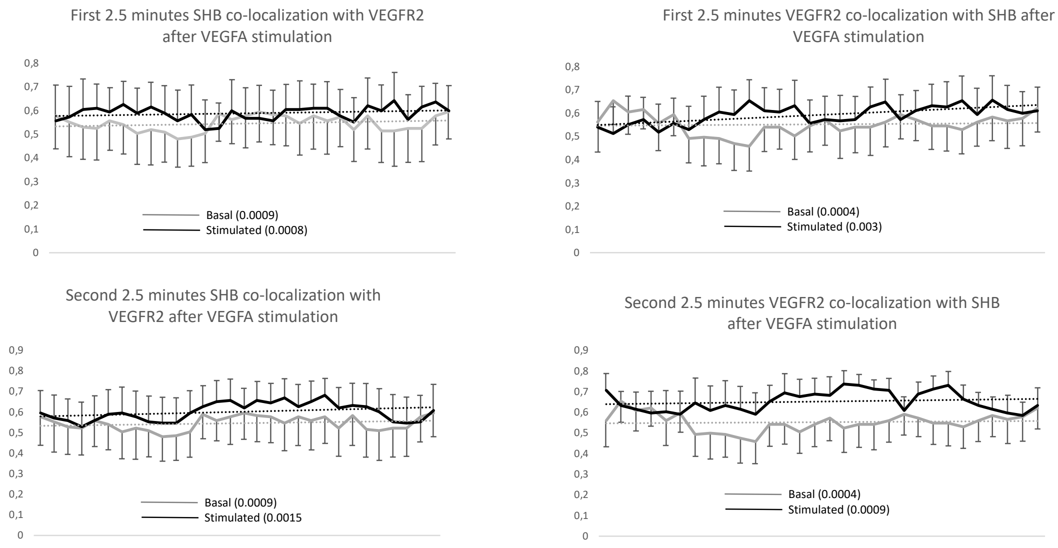

Supplemental Figure 1: TIRF co-localization as indicated. Co-localization at each time point was determined for each recording (individual observation) and means ± SEM for the individual time point values were calculated and plots generated for each experimental condition. These were used to obtain trendlines (trendlines = slopes) that are shown with values indicated in the legends. These will be different from the ones shown in Figures 2-4 since the latter are means of individual trendlines/slopes, each obtained from a separate recording. n=6.

Supplemental Figure 2

HEK293 cells and SHB/Y951F-VEGFR2

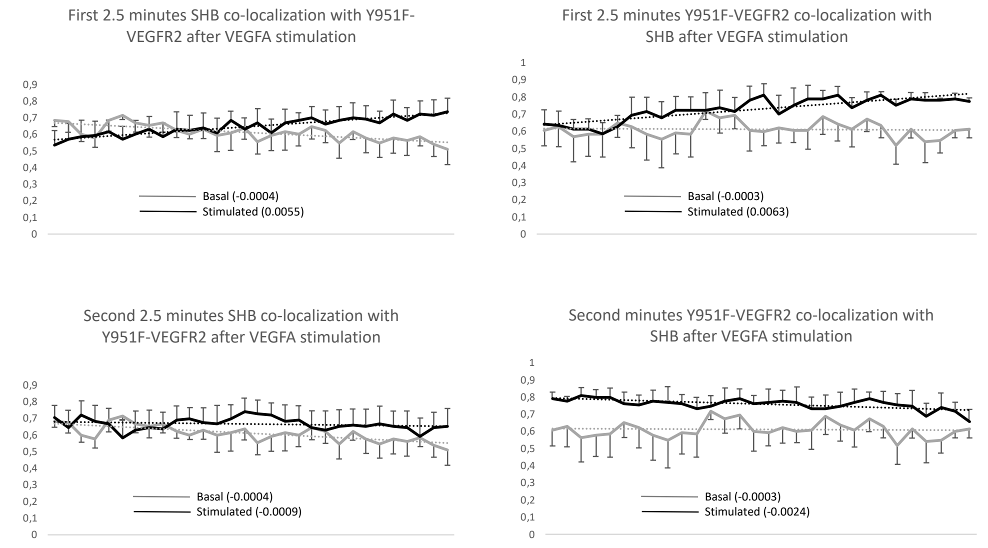

Supplemental Figure 2: TIRF co-localization as described in S. Fig. 1 with trendlines (=slopes) indicated. n= 6.

Supplemental Figure 3

HEK293 cells and SHB/Y1175F-VEGFR2

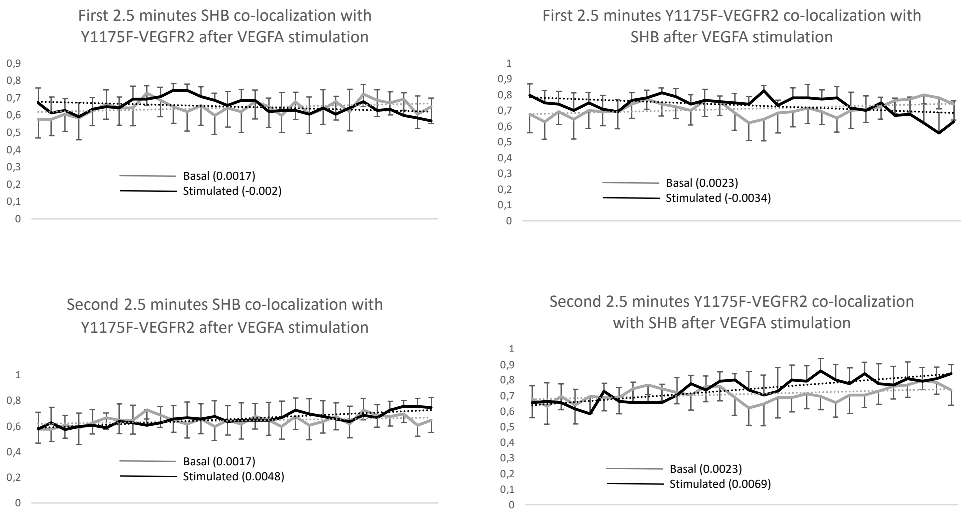

Supplemental Figure 3: TIRF co-localization as indicated in S. Fig. 1 with trendlines (=slopes) indicated. n=6.

Supplemental Figure 4

Wild type EC and VEGFR2/FAK

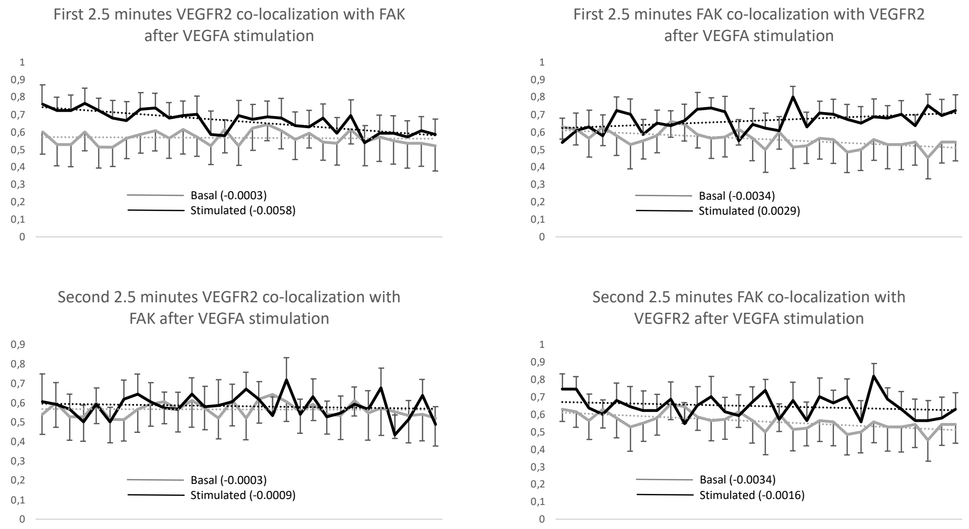

Supplemental Figure 4: TIRF co-localization as indicated in S. Fig. 1. Trendlines (=slopes) were determined as indicated in the legends and will be different from the ones shown in Figure 4 since the latter are means of individual trendlines for each recording whereas the trendlines of this figure are based on pooled co-localization data for each time point. n=6.

Supplemental Figure 5

Shb KO EC and VEGFR2/FAK

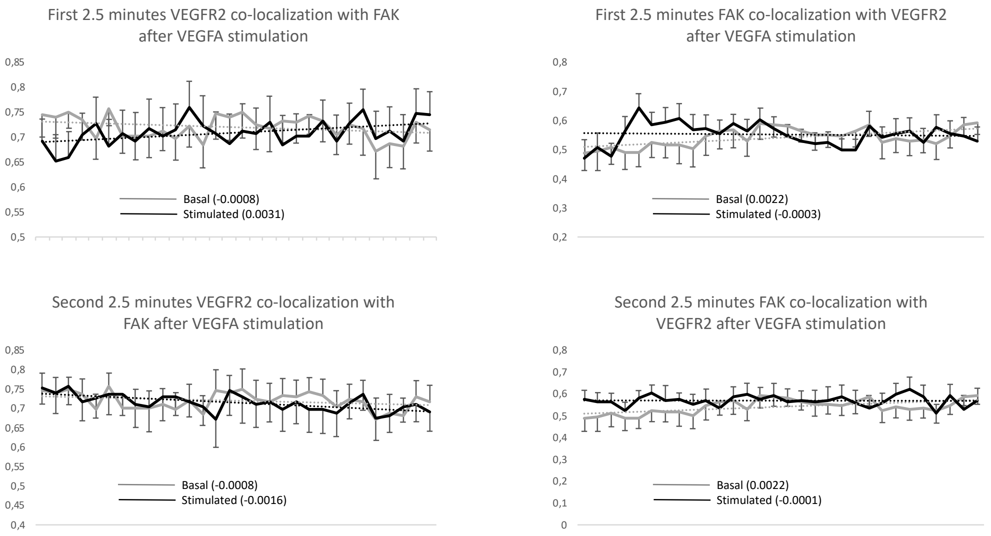

Supplemental Figure 5: TIRF co-localization as indicated in S. Fig. 1. Trendlines (=slopes) are given. n=5.

Supplemental Figure 6:

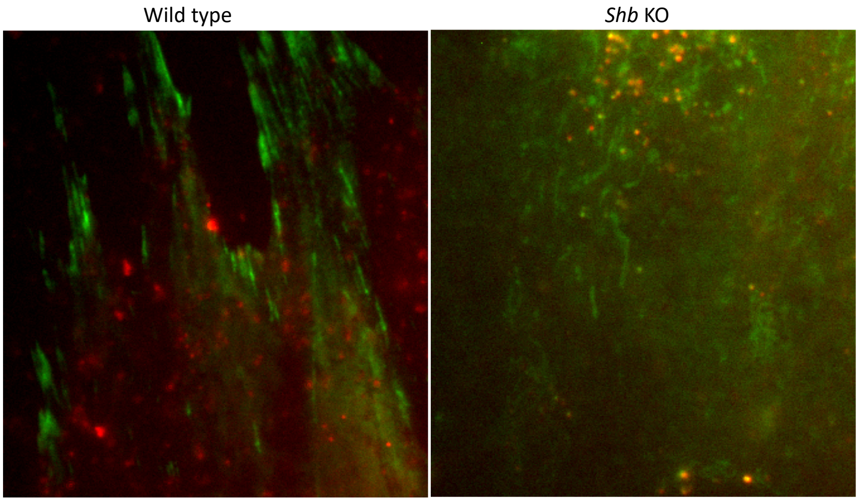

Supplemental Figure 6: TIRF snapshots of mEmerald-FAK (green) and mChreey-VEGFR2 (red) fluorescence after transfection to wild type and *Shb* KO EC. Note the perinuclear localization of mEmerald-FAK in the KO situation that resembles the corresponding paxillin staining pattern whereas the wild type cells display peripheral localization.

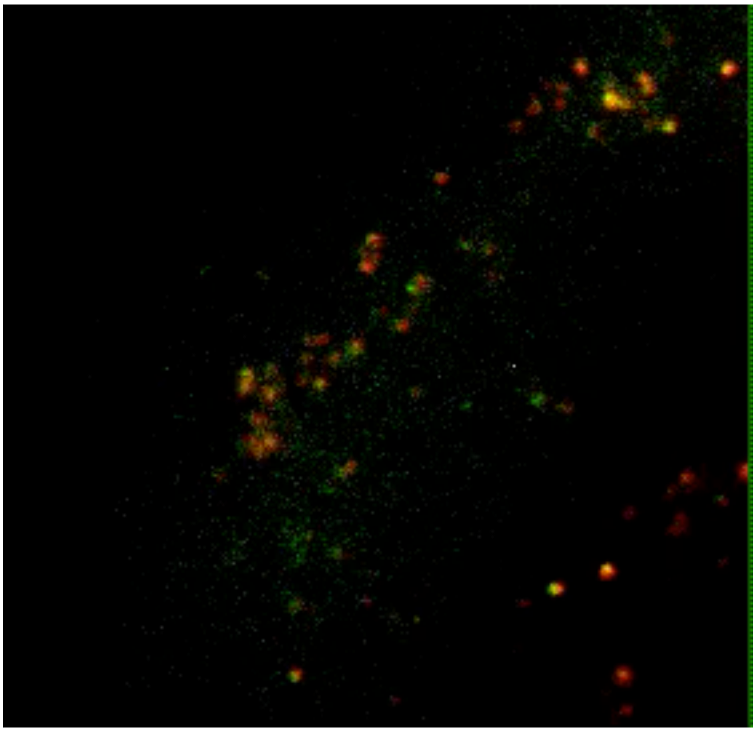

Supplemental Movie 1: Example of TIRF recording of EC transfected with mEmerald-FAK (green) and mCherry-VEGFR2 (red) (co-localization yellow) for 2.5 minutes prior to VEGFA addition followed by 2.5 minutes with VEGFA. Please note that the changes in trendlines cannot be seen visually and only detected by numerical analysis in Excel.
